# Interleukin-22 Attenuated Renal Tubular Injury in Aristolochic Acid Nephropathy via Suppressing Activation of NLRP3 Inflammasome

**DOI:** 10.3389/fimmu.2019.02277

**Published:** 2019-09-24

**Authors:** Shaofei Wang, Jiajun Fan, Xiaobin Mei, Jingyun Luan, Yubin Li, Xuyao Zhang, Wei Chen, Yichen Wang, Guangxun Meng, Dianwen Ju

**Affiliations:** ^1^Minhang Hospital, Fudan University, Shanghai, China; ^2^School of Pharmacy, Fudan University, Shanghai, China; ^3^Department of Nephrology, Changhai Hospital, Second Military Medical University, Shanghai, China; ^4^Unit of Innate Immunity, Key Laboratory of Molecular Virology and Immunology, Institute Pasteur of Shanghai, Shanghai Institutes for Biological Sciences, Chinese Academy of Sciences, Shanghai, China

**Keywords:** IL-22, aristolochic acid nephropathy, NLRP3 inflammasome, tubular injury, renal function

## Abstract

Aristolochic acid nephropathy (AAN), as a rapidly progressive interstitial nephropathy due to excessive ingestion of aristolochia herbal medications, has recently raised considerable concerns among clinicians and researchers as its underlying pathogenic mechanisms are largely unclear. In the current study, we identified NLRP3 inflammasome activation as a novel pathological mechanism of AAN. We found that NLRP3 inflammasome was aberrantly activated both *in vivo* and *in vitro* after AA exposure. Blockade of IL-1β and NLRP3 inflammasome activation by IL-1Ra significantly attenuated renal tubular injury and function loss in AA-induced nephropathy. Moreover, NLRP3 or Caspase-1 deficiency protected against renal injury in the mouse model of acute AAN, suggesting that the NLRP3 signaling pathway was probably involved in the pathogenesis of AAN. We also found that administration of IL-22 could markedly attenuate renal tubular injury in AAN. Notably, IL-22 intervention significantly alleviated renal fibrosis and dysfunction in AA-induced nephropathy. Furthermore, IL-22 largely inhibited renal activation of NLRP3 inflammasome in AA-induced nephropathy. These results indicated that IL-22 ameliorated renal tubular injury in AAN through suppression of NLRP3 inflammasome activation. In summary, this study identified renal activation of NLRP3 inflammasome as a novel mechanism underlying the pathogenesis of AAN, thus providing a potential therapeutic strategy for AAN based on suppression of NLRP3 inflammasome activation.

## Introduction

Aristolochic acid nephropathy (AAN), characterized pathologically by acute tubular injury and clinically by irreversible renal function deterioration, is a progressive tubulointerstitial nephritis that will ultimately lead to end-stage renal disease (ESRD) and urothelial malignancy if uncontrolled (1–3). In recent years, AAN has been recognized as a worldwide public health problem with its actual incidence probably underestimated. However, despite tubular epithelial cells being identified as the primary targets in AAN (4, 5), current understandings of the pathologic mechanism of AAN are still inadequate. Therefore, to develop novel therapeutic strategies for AAN, further investigations into the molecular mechanism of the pathogenesis of the disease are still urgently needed.

The NOD-like receptor family pyrin domain-containing protein 3 (NLRP3) inflammasome (6, 7), a crucial contributor to tissue injury via converting downstream inflammatory cytokines IL-1β and IL-18 into their corresponding active forms, has been increasingly implicated in the pathogenesis of various kidney diseases, including both tubulointerstitial and glomerular diseases (8). It has been indicated that renal activation of the NLRP3 inflammasome not only occurred in infiltrating immunocytes, primarily including macrophages and dendritic cells, but also in some intrinsic renal cells such as kidney tubular epithelial cells. In recent years, renal tubular epithelial cells have intensively been demonstrated to express the components of the NLRP3 inflammasome (9–12). Moreover, there is growing evidence that renal NLRP3 inflammasome activation contributes to tubular cell damage in acute kidney injury (13, 14). NLRP3 inflammasome mediated tubular injury and dysfunction by exerting a direct effect on the renal tubular epithelium in renal ischemic acute tubular necrosis (15). However, despite numerous literatures demonstrating a detrimental role of NLRP3 inflammasome in experimental renal injury, whether the activation of NLRP3 inflammasome is associated with the progression of AAN remains largely undetermined. The role of NLRP3 signaling pathway in the development and progression of ANN have not been previously described.

IL-22, as a member of the IL-10 superfamily exerting its biological effects via binding to IL-22R1/IL-10R2 transmembrane receptor complex (16, 17), has been increasingly implicated in the prevention of renal tissue damage (18–20). Given that the responsiveness to IL-22 was mainly determined by the existence of IL-22R1 which was primarily expressed in the proximal tubules (21, 22), IL-22 specifically targeted the kidney proximal tubular epithelial cells but not the other types of cells within kidney tissues. Notably, there is a growing evidence demonstrating that IL-22 possesses a therapeutic potential for acute kidney injury by targeting renal tubular epithelium. Previous literatures have indicated that IL-22 efficiently ameliorated renal lesions, accelerated renal regeneration and preserved renal function in acute renal injury through downregulation of the inflammatory responses (18, 19, 23). It has also been reported that IL-22 could restrain NLRP3 inflammasome activation by facilitating the sustained generation of the IL-1 receptor antagonist (IL-Ra) (24). Moreover, our recent study has demonstrated that IL-22 could ameliorate renal lesion and fibrosis in diabetic kidney disease by suppression of renal NLRP3 inflammasome activation (20). However, it still remains undetermined whether IL-22 could attenuate AA-induced renal tubular injury through downregulation of NLRP3 inflammasome activation.

In the current study, we intended to decipher the role of renal NLRP3 inflammasome activation in the pathogenic mechanisms of AA-induced tubular injury and evaluate the therapeutic potential of IL-22 for AAN through regulation of the NLRP3 inflammasome activation. This study demonstrated that blockage of NLRP3 inflammasome activation or its downstream pro-inflammatory cytokines release might be a novel therapeutic strategy for AAN.

## Materials and Methods

### Materials and Reagents

The aristolochic acid I sodium salt used to generate mouse model of acute AAN was purchased from Sigma (St. Louis, MO, USA). The recombinant mouse IL-22 was obtained from Novoprotein (Shanghai, China) and the recombinant human IL-1Ra was kindly provided by General Regeneratives Limited (Shanghai, China). The primary and secondary antibodies used for immunoblot analysis were as follows: anti-Fibronectin (ab2413) and anti-Collagen IV (ab6586) from Abcam (Cambridge, Massachusetts, USA); anti-α-Smooth Muscle Actin (α-SMA) (14968), anti-Vimentin (D21H3) (5741), anti-NLRP3 (15101), anti-Cleaved Caspasse-1 (67314), anti-IL-1β (12242), and anti-β-actin (8H10D10) (3700) from Cell Signaling Technology (Danvers, MA, USA); peroxidase-conjugated goat anti-rabbit and anti-mouse immunoglobulin G (IgG) from Jackson ImmunoResearch Laboratory (West Grove, PA, USA). The primary and secondary antibodies used for immunohistochemical staining of NLRP3 were as follows: anti-NLRP3 (GB11300) and HRP-conjugated goat anti-rabbit IgG (GB23303) from Servicebio Technology Co. Ltd. (Wuhan, China).

### Mouse Models of Acute AAN (25) and *in vivo* Intervention Studies

In the current study, AAN was induced in 8-week-old male BALB/c mice from SLAC Laboratory Animal Co. Ltd. (Shanghai, China) via a daily intraperitoneal injection of AAI sodium salt (5 mg/kg body weight dissolved in sterile water) or 8-week-old male C57BL/6 mice from SLAC Laboratory Animal Co. Ltd. (Shanghai, China) by a daily intraperitoneal injection of AAI sodium salt (10 mg/kg body weight dissolved in sterile water) for 5 consecutive days. Age-matched mice intraperitoneally injected with saline instead of AAI sodium salt for 5 consecutive days were served as controls.

To determine whether IL-1Ra intervention could prevent the progression of AAN through blockade of IL-1β and NLRP3 inflammasome activation, the recombinant IL-1Ra (10 mg/kg body weight) was injected intraperitoneally 1 h before each AA exposure for 5 consecutive days in mouse model of acute AAN generated by both BALB/c (*N* = 6 per group) and C57BL/6 (*N* = 6 per group) mice as was described above. To determine whether IL-22 intervention could prevent the progression of AAN through inhibition of NLRP3 inflammasome activation, the recombinant IL-22 (100 μg /kg body weight) was injected intraperitoneally 1 h before each AA exposure for 5 consecutive days in a mouse model of acute AAN generated by both BALB/c (*N* = 6 per group) and C57BL/6 mice (*N* = 7 per group) as was described above.

To determine whether NLRP3 or Caspase-1 was involved in the pathogenesis of acute AAN, NLRP3^−/−^, or Caspase-1^−/−^ together with age-matched wild-type male BALB/c mice were exposed to AA by a daily intraperitoneal injection of AAI sodium salt (5 mg/kg body weight dissolved in sterile water) for 5 consecutive days (*N* = 3 per group). NLRP3-deficient mice were generated as previously described (26, 27) and Caspase-1-deficient mice were obtained from Jackson Laboratory. Wild-type BALB/c mice were purchased SLAC Laboratory Animal Co. Ltd. (Shanghai, China). NLRP3- and Caspase-1-deficient mice were crossed with BALB/c for at least 10 generations before the initiation of the current experiment (28). All the *in vivo* experimental procedures were performed according to the standards approved by Animal Ethical Committee of School of Pharmacy at Fudan University.

### Renal Histopathological and Immunohistochemical Analysis

The mice were sacrificed under anesthesia (sodium pentobarbital, 50 mg/kg body weight, intraperitoneally) 24 h after the last AA exposure and the kidney tissues were collected, weighted, and immediately processed for H&E staining and then subjected to histopathological analysis. Renal tubular injury in AAN, characterized by desquamation of necrotic tubular epithelial cells, loss of brush border, dilation of tubules, and formation of cellular casts, was semi-quantified by the percentage of tubules area that displayed lesions mentioned above according to the following grading scale: Score 0 = none; Score 1 ≤ one-third; Score 2 ≤ two-thirds; Score 3 ≤ 100%. Briefly, to quantify the severity of renal tubular injury in AAN, 10 fields under 200-fold magnification from each slide were randomly selected and scored by two investigators independently in a blinded fashion. Immunohistochemical staining for NLRP3 with Harris hematoxylin as counterstaining for nuclei was performed to determine the localization and expression of NLRP3 in renal tissues. Briefly, NLRP3 negative and positive tubules were counted manually within at least 10 non-overlapping regions at a magnification of 400-fold of each slide by cell counter plugins of ImageJ 1.49v (National Institutes of Health, Bethesda, MD, USA) and the percentage of NLRP3 positive tubules was used to quantify the expression of NLRP3 in renal tissues.

### Renal Function and Caspase-1 Activity Assay

The non-fasting blood samples were drawn by retro-orbital bleeding and 6-hour urine samples were harvested by metabolic cages 4 days after AA exposure to determine the serum and urinary biochemical parameters of the mice. Serum creatinine, blood urea nitrogen (BUN), proteinuria and urinary N-acetyl-β-d-glucosaminidase (NAG) were determined to evaluate renal function using commercially available detection kit from Nanjing Jiancheng Bioengineering Institute (Nanjing, China) in accordance with the manufacture's instruction. Renal index was calculated by renal weight (mg)/body weight (g). The renal Caspase-1 activity was determined by an Caspase-1 Activity Assay Kit from Beyotime Institute of Biotechnology (Beijing, China) and normalized to the protein content as previously described (20).

### Cytokine Level Assay

Cytokine levels of murine IL-22 in serum and renal homogenates of mice were determined by ELISA kit from Multi Sciences (Hangzhou, China) according to the manufacturer's instructions. Serum levels of murine IL-1β and IL-18 were determined by ELISA kits from Boatman Biotech (Shanghai, China) and Multi Sciences (Hangzhou, China), respectively.

### Renal Tubular Epithelial Cell Culture and *in vitro* Experiment

Human renal proximal tubular epithelial cells HK-2 were purchased and cultured as previously described (20). To explore the effect of IL-22 on AA-induced NLRP3 inflammasome activation in renal tubular epithelial cells, HK-2 cells were stimulated with or without AAI sodium salt (10 μg/mL) in the absence or presence of various concentrations of murine IL-22 for 24 h as indicated. And then HK-2 cells subjected to different treatments were collected, lysed with cell lysis buffer and processed for immunoblot analysis.

### Immunoblot Analysis

Equal amounts of total proteins extracted from cultured cells or mouse kidney tissues were subjected to SDS-PAGE followed by immunoblot analysis. The densitometric value of each immunoreactive band was semi-quantified, normalized to that of β-actin using ImageJ 1.49v and expressed as fold change.

### Statistical Analysis

All the data in this study were presented as the mean ± standard error of the mean (SEM). Statistical analysis was performed by Student's *t*-test for comparison between two groups and one-way analysis of variance (ANOVA) for comparison among multiple groups followed by nonparametric test using GraphPad Prism 6.0 Software (GraphPad Software Inc., San Diego, CA, USA).

## Results

### Activation of NLRP3 Inflammasome Was Aberrantly Induced in AAN

**Figure 1:**
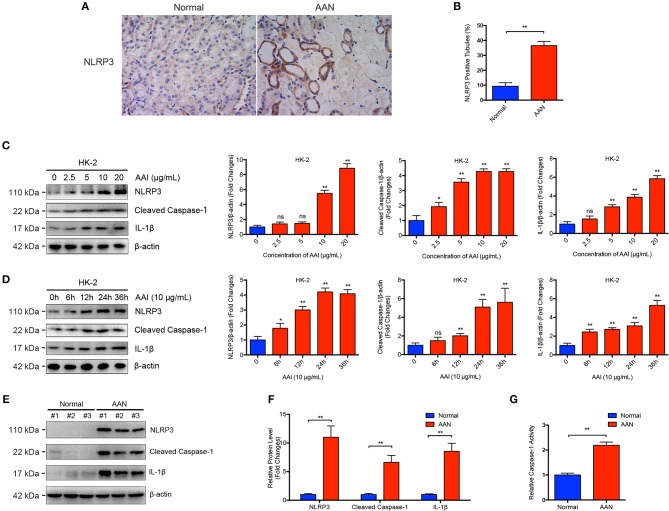
Aberrant activation of NLRP3 inflammasome in acute AAN. **(A)** Representative immunohistochemical staining of NLRP3 in renal tissues in acute AAN. AAN was induced in BALB/c mice by a daily intraperitoneal injection of AAI sodium salt (5 mg/kg body weight) for 5 consecutive days. **(B)** Semi-quantitative analysis of NLRP3 expression in renal tissues. The data were expressed as the percentage of NLRP3 positive tubules in mouse model of acute AAN generated by BALB/c mice. **(C)** Representative immunoblots and semi-quantification of NLRP3, cleaved form of Caspase-1, and IL-1β expression in HK-2 cells after exposed to various concentrations of AAI sodium salt (0, 2.5, 5, 10, 20 μg/mL) for 24 h from 3 independent experiments. **(D)** Representative immunoblots and semi-quantification of NLRP3, cleaved form of Caspase-1, and IL-1β expression in HK-2 cells after exposed to 10 μg/mL of AAI sodium salt for indicated times (0, 6, 12, 24, 36 h) from 3 independent experiments. **(E)** Representative immunoblots of renal NLRP3, cleaved form of Caspase-1, and IL-1β expression in mouse model of acute ANN. AAN was induced in C57BL/6 mice by a daily intraperitoneal injection of AAI sodium salt (10 mg/kg body weight) for 5 consecutive days. **(F)** Semi-quantification of renal NLRP3, cleaved form of Caspase-1, and IL-1β expression in mouse model of acute ANN generated by C57BL/6 mice (*N* = 6). **(G)** Relative activity of renal cytosolic Caspase-1 in mouse model of acute AAN generated by C57BL/6 mice after AA exposure (*N* = 6). ns = no significance; ^*^p < 0.05; ^**^p < 0.01.

NLRP3 inflammasome activation and subsequent IL-1β maturation have increasingly been reported to participate in the pathogenesis of various kidney diseases. Renal immunohistochemical analysis revealed that renal NLRP3 expression was substantial elevated in AAN, with the positive staining mainly localized in damaged renal tubular epithelial cells but not renal interstitial cells (Figures 1A,B). After aristolochic acid exposure, only the damaged renal tubules exhibited significant upregulated expression of NLRP3 while normal renal tubules exhibited basal level of NLRP3 expression, indicating a possible causal link between elevated NLRP3 expression and aggregated tubular injury in AAN. To further explore whether renal NLRP3 inflammasome was aberrantly activated in AAN, we determined the expression of NLRP3 inflammasome components after AA exposure both *in vivo* and *in vitro* by immunoblot analysis. As shown in Figures 1C,D, exposure of renal proximal tubular epithelial cells to AA triggered a dose- and time-dependent activation of NLRP3 inflammasome, as demonstrated by elevated expression of NLRP3, Cleaved Caspase-1, and its downstream cytokine IL-1β. Meanwhile, renal activation of the NLRP3 inflammasome was remarkably upregulated in a mouse model of acute AAN, as indicated by overexpression of components of NLRP3 inflammasome (Figures 1E,F). In addition, it was indicated that renal Caspase-1 activity was also markedly upregulated in mice with acute AAN (Figure 1G). These data demonstrated that the NLRP3 inflammasome was abnormally activated in AAN.

### Blockade of IL-1β and NLRP3 Inflammasome Activation by IL-1Ra Alleviated Renal Injury and Function Loss in AAN

**Figure 2:**
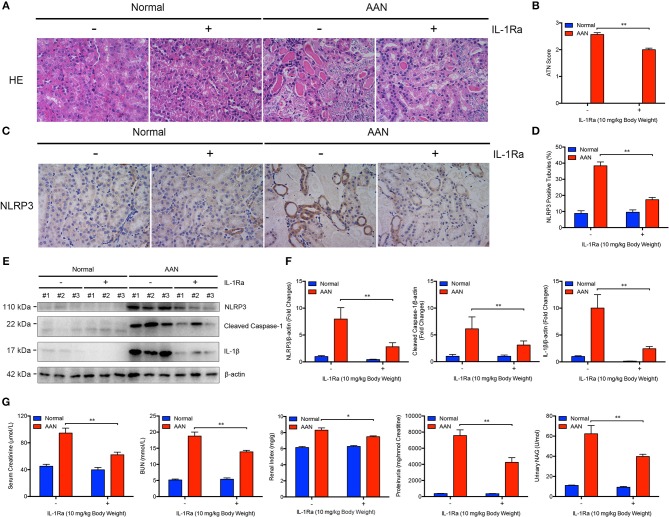
Alleviated AA-induced renal injury and function loss after blockade of IL-1β and NLRP3 inflammasome activation by IL-1Ra. **(A)** Representative micrographs of renal tubular injury detected by H&E staining in acute AAN after IL-1Ra intervention. AAN was induced in BALB/c mice by a daily intraperitoneal injection of AAI sodium salt (5 mg/kg body weight) for 5 consecutive days with the recombinant IL-1Ra (10 mg/kg body weight) injected intraperitoneally 1 h before each AA exposure. Original magnification: 400×. **(B)** Semi-quantification of the severity of renal tubular injury. The ATN score in mouse model of acute AAN generated by BALB/c mice after IL-1Ra intervention was rated by histopathological analysis from H&E staining (*N* = 3). **(C)** Representative immunohistochemical staining of NLRP3 in renal tissues in mouse model of acute AAN generated by BALB/c mice after IL-1Ra intervention. Original magnification: 400×. **(D)** Semi-quantitative analysis of NLRP3 expression in renal tissues in mouse model of acute AAN generated by BALB/c mice after IL-1Ra intervention. The data were expressed as the percentage of NLRP3 positive tubules (*N* = 3). **(E)** Representative immunoreactive bands and **(F)** densitometric analysis of renal NLRP3, cleaved form of Caspase-1, and IL-1β expression of acute AAN mice after IL-1Ra intervention (*N* = 3). AAN was induced in C57BL/6 mice by a daily intraperitoneal injection of AAI sodium salt (10 mg/kg body weight) for 5 consecutive days with the recombinant IL-1Ra (10 mg/kg body weight) injected intraperitoneally 1 h before each AA exposure. **(G)** Serum creatinine, BUN, renal index, proteinuria, and urinary NAG levels in mouse model of acute AAN generated by C57BL/6 mice after IL-1Ra intervention (*N* = 6). ^*^p < 0.05; ^**^p < 0.01.

To explore the critical role of NLRP3 inflammasome and its major downstream cytokine IL-1β in the pathogenesis of AAN, IL-1Ra was applied to block NLRP3 inflammasome activation and IL-1β activity in AA-induced nephropathy. We found that consecutive AA exposure led to obvious tubular injury, as indicated by numerous exfoliative necrotic tubular epithelial cells and dilated tubules. In contrast, IL-1Ra markedly alleviated AA-induced acute renal tubular injury (Figures 2A,B). Moreover, renal immunohistochemical analysis revealed that IL-Ra could largely downregulate the expression of NLRP3 in renal tubules as well as tubular injury in AAN (Figures 2C,D). Simultaneously, administration of IL-1Ra significantly suppressed renal overexpression of NLRP3, Cleaved Caspase-1, and mature IL-1β induced by AA exposure, indicating reduction of renal activation of NLPR3 inflammasome by IL-1Ra in AAN (Figures 2E,F). Additionally, pharmacological inhibition of IL-1 receptor signaling by IL-1Ra largely downregulated markers of the severity of acute kidney injury such as serum creatinine, BUN, renal index, proteinuria secretion, and urinary NAG, suggesting restoration of renal function by IL-1Ra in acute AAN (Figure 2G). These data indicated that blockade of IL-1β and NLRP3 inflammasome activation by IL-1Ra remarkably attenuated AA-induced renal tubular injury and function loss.

### NLRP3 or Caspase-1 Deficiency Protected Against Renal Injury in AAN

**Figure 3:**
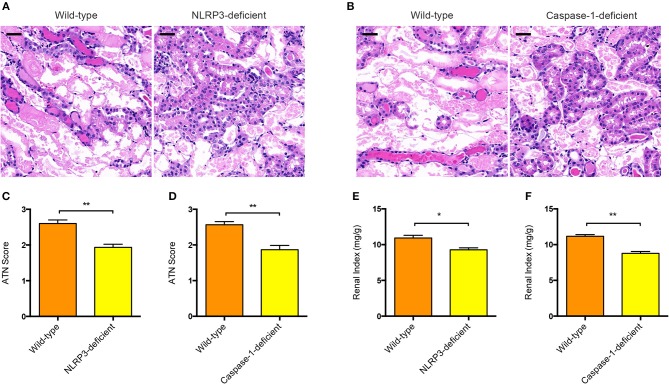
Involvement of NLRP3 or Caspase-1 in the pathogenesis of AAN. **(A)** Representative micrographs of renal tubular injury detected by H&E staining in wild-type and NLRP3-deficient mice (BALB/c background) after AA exposure. Scale bar = 100 μm. **(B)** Representative micrographs of renal tubular injury detected by H&E staining in wild-type and Caspase-1-deficient mice (BALB/c background) after AA exposure. Scale bar = 100 μm. **(C)** Semi-quantification of the severity of renal tubular injury. The ATN score in wild-type and NLRP3-deficient mice after AA exposure was rated by histopathological analysis from H&E staining (*N* = 3). **(D)** Semi-quantification of the severity of renal tubular injury. The ATN score in wild-type and Caspase-1-deficient mice after AA exposure was rated from H&E staining micrographs (*N* = 3). **(E)** Renal index of NLRP3-deficient and wild-type mice with acute AAN (*N* = 3). **(F)** Renal index of Caspase-1-deficient and wild-type mice with acute AAN (*N* = 3). ^*^p < 0.05; ^**^p < 0.01.

To further determine the functional relevance of NLRP3 inflammasome activation with pathogenesis of AAN, we induced acute AAN in NLRP3^−/−^ or Caspase-1^−/−^ mice together with wild-type mice by intraperitoneal injection of AA for 5 consecutive days. As was shown evidently in Figures 3A,C, NLRP3-deficient mice exhibited attenuated renal tubular injury after acute AA exposure compared with wild-type mice. In parallel, Caspase-1 deficiency also protected against AA-induced renal injury (Figures 3B,D), indicating that the NLRP3/Caspase-1 signaling pathway was involved in the progression of AAN. Besides, renal index of NLRP3^−/−^ or Caspase-1^−/−^ mice exposed to AA was significantly downregulated as compared to that of wild-type controls (Figures 3E,F). Collectively, these data strongly demonstrated that NLRP3 inflammasome activation probably played a fundamental role in the pathogenesis of AAN.

### IL-22 Attenuated Renal Injury and Fibrosis in AAN

**Figure 4:**
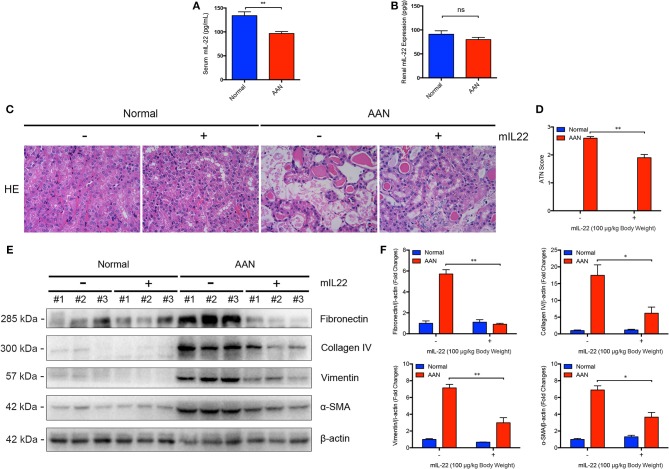
Attenuated renal injury and fibrosis mediated by IL-22 in AAN. **(A)** Serum levels of endogenous murine IL-22 in mouse model of acute AAN generated by BALB/c mice (*N* = 8). **(B)** The endogenous IL-22 levels in renal homogenates in mouse model of acute AAN generated by BALB/c mice (*N* = 8). **(C)** Representative micrographs of renal tubular injury detected by H&E staining in acute AAN after IL-22 intervention. AAN was induced in BALB/c mice by a daily intraperitoneal injection of AAI sodium salt (5 mg/kg body weight) for 5 consecutive days with the recombinant IL-22 (100 μg/kg body weight) injected intraperitoneally 1 h before each AA exposure. Original magnification: 400×. **(D)** Semi-quantification of the severity of renal tubular injury. The ATN score in mouse model of acute AAN generated by BALB/c mice after IL-22 intervention was rated by histopathological analysis from H&E staining (*N* = 3). **(E)** Renal expression of fibrosis markers including Fibronectin, Collagen IV, Vimentin, and α-SMA in mouse model of acute AAN generated by C57BL/6 mice after IL-22 intervention. **(F)** Densitometric analysis of renal fibrosis markers expression such as Fibronectin, Collagen IV, Vimentin, and α-SMA in mouse model of acute AAN generated by C57BL/6 mice after IL-22 intervention (*N* = 3). ^*^p < 0.05; ^**^p < 0.01.

To explore the possible alteration of endogenous IL-22 expression in AAN, we determined the murine IL-22 levels in serum and renal homogenates by ELISA assay. We found that the serum level of IL-22 in mice was markedly downregulated after consecutive AA exposure (Figure 4A). Meanwhile, renal IL-22 expression in AA-induced nephropathy was slightly but not significantly downregulated (Figure 4B), indicating possible downregulation of IL-22 in AAN. Our previous study had demonstrated that IL-22 could alleviate renal lesion and fibrosis in diabetic kidney disease through suppression of NLRP3 inflammasome activation (20). Given that NLRP3 inflammasome activation contributed to the progression of AAN, targeting the NLRP3 signaling pathway might be a feasible therapeutic strategy for AAN. In this study, to determine whether intervention by IL-22 could exert similar effects on AA-induced nephropathy, IL-22 was administrated intraperitoneally once a day before each AA exposure for 5 consecutive days. As was elucidated by Figures 4C,D, consecutive AA exposure resulted in severe renal tubular injury, whereas IL-22 intervention significantly attenuated renal lesion induced by acute AA exposure. Meanwhile, renal expression of fibrosis markers such as Fibronectin, Collagen IV, Vimentin, and α-SMA were markedly upregulated after AA exposure (Figures 4E,F). In contrast, administration of IL-22 significantly downregulated the renal overexpression of Fibronectin, Collagen IV, Vimentin, and α-SMA, suggesting that IL-22 intervention could alleviate renal fibrosis in AAN. Collectively, these data indicated that IL-22 could attenuate renal tubular lesion and fibrosis in AA-induced nephropathy.

### IL-22 Alleviated Renal Dysfunction in Acute AAN

**Figure 5:**
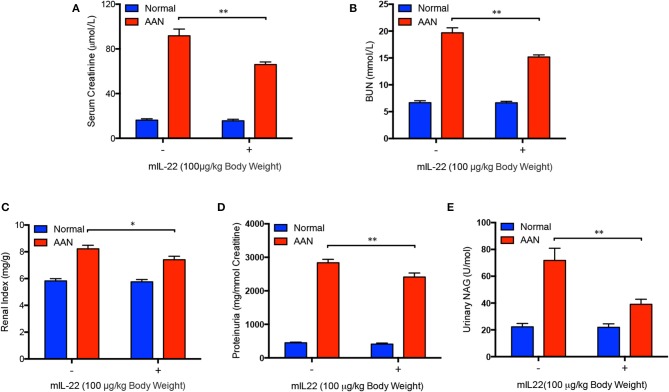
Improved renal function after IL-22 intervention in acute AAN. **(A)** Serum levels of creatinine, **(B)** BUN, **(C)** renal index, **(D)** proteinuria, and **(E)** urinary NAG levels in mouse model of acute AAN generated by C57BL/6 mice after IL-22 intervention. *N* = 7; ^*^p < 0.05; ^**^p < 0.01.

To determine whether IL-22 could improve renal function in mice with acute AAN, a series of frequently used markers of renal injury and function were also measured after IL-22 intervention. As it was shown in Figures 5A,B, serum creatinine and BUN levels were both obviously upregulated after AA exposure. In contrast, serum levels of creatinine in mice with acute AAN were significantly downregulated by IL-22 intervention. Meanwhile, IL-22 intervention led to a marked reduction of BUN levels in mice exposed to AA, indicating largely improved renal function induced by IL-22 in AAN. Renal index was also drastically increased in AA-induced nephropathy but significantly decreased by IL-22 intervention (Figure 5C). Furthermore, administration of IL-22 largely reduced proteinuria secretion in acute AAN (Figure 5D). In addition, urinary NAG, as a marker of kidney tubular dysfunction, was obviously downregulated by IL-22 in AA-induced nephropathy (Figure 5E). Taken together, these findings demonstrated that IL-22 could efficiently attenuate renal dysfunction in acute AAN.

### Nephroprotective Role of IL-22 in AAN Was Probably Mediated by Downregulation of NLRP3 Inflammasome Activation

**Figure 6:**
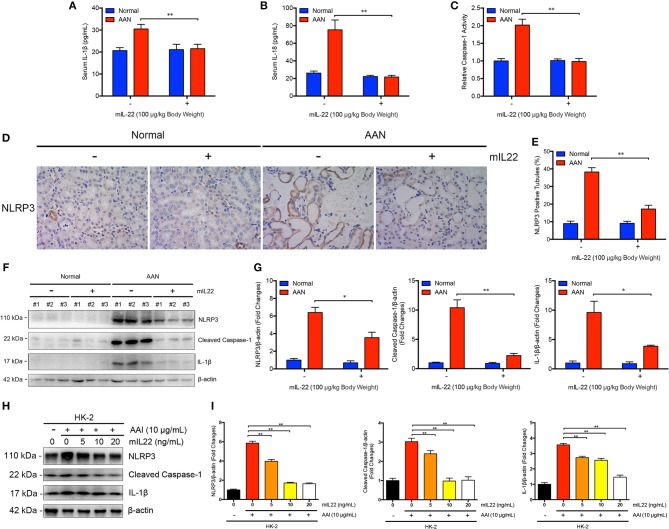
Inhibition of renal NLRP3 inflammasome activation mediated by IL-22 in AA-induced renal injury. **(A)** Serum levels of murine IL-1β and **(B)** IL-18 in mouse model of acute ANN generated by C57BL/6 mice after IL-22 intervention measured by ELISA (*N* = 7). **(C)** Relative activity of renal cytosolic Caspase-1 in mouse model of acute ANN generated by C57BL/6 mice after IL-22 intervention (*N* = 7). **(D)** Representative immunohistochemical staining of NLRP3 in renal tissues in mouse model of acute AAN generated by BALB/c mice after IL-22 intervention. Original magnification: 400×. **(E)** Semi-quantitative analysis of NLRP3 expression in renal tissues in mouse model of acute AAN generated by BALB/c mice after IL-22 intervention. The data were expressed as the percentage of NLRP3 positive tubules (*N* = 3). **(F)** Representative immunoreactive bands and **(G)** densitometric analysis of renal NLRP3, cleaved form of Caspase-1, and IL-1β expression in mouse model of acute AAN generated by C57BL/6 mice after IL-22 intervention (*N* = 3). **(H)** Representative immunoreactive bands and **(I)** densitometric analysis of NLRP3, cleaved form of Caspase-1, and IL-1β expression in HK-2 cells after AA exposure (10 μg/mL) with or without IL-22 intervention (0, 5, 10, 20 ng/mL) from 3 independent experiments (*N* = 3). ^*^p < 0.05; ^**^p < 0.01.

Next, we explored whether IL-22 exerted favorable effects in acute AAN through inhibition of renal activation of NLRP3 inflammasome. As evidenced by Figures 6A,B, murine IL-1β and IL-18 levels in serum of mice were both significantly upregulated after consecutive AA exposure. In contrast, serum murine IL-1β and IL-8 levels were remarkably downregulated in AAN after IL-22 intervention, suggesting systemic anti-inflammatory effects of IL-22 in AA-induced nephropathy. Notably, administration of IL-22 normalized elevated renal Caspase-1 activity induced by AA exposure as evidenced by Caspase-1 activity assay (Figure 6C). Renal immunohistochemical analysis revealed that IL-22 substantially downregulated the expression of NLRP3 in renal tubules as well as tubular injury in AAN (Figures 6D,E). In parallel, immunoblot analysis demonstrated that consecutive AA exposure led to drastic renal NLRP3 inflammasome activation, subsequent Caspase-1 cleavage and IL-1β maturation, whereas IL-22 intervention markedly downregulated renal overexpression of NLRP3 inflammasome components in AAN, indicating potent anti-inflammatory effects of IL-22 through suppression of NLRP3 inflammasome activation *in vivo* (Figures 6F,G). Moreover, IL-22 dose-dependently inhibited AA-induced aberrant NLRP3 inflammasome activation in human proximal epithelial cells, suggesting that IL-22 could suppress renal activation of NLRP3 inflammasome at least partially by directly targeting tubular epithelial cells (Figures 6H,I). These findings demonstrated that IL-22 could alleviate kidney injury in AAN through inhibition of renal activation of NLRP3 inflammasome. In summary, we found that the activation of NLRP3 inflammasome participated in the development and progression of AA-induced nephropathy and IL-22 exerted a nephroprotective effect by suppressing renal NLRP3 inflammasome activation.

## Discussion

This study revealed a novel correlation between activation of NLRP3 inflammasome and the progression of AAN. In the current study, we reported for the first time that NLRP3 inflammasome was aberrantly activated *in vitro* and *in vivo* after AA exposure. Notably, we demonstrated that NLRP3 or Caspase-1 deficiency ameliorated renal tubular injury in a murine model of AAN, thus identifying renal NLRP3 inflammasome activation as a crucial mediator in the pathogenesis of AA-induced nephropathy. Furthermore, on the basis of elucidating a possible association between the activation of NLRP3 inflammasome and the pathogenesis of AAN, we also demonstrated for the first time that administration of IL-22 could attenuate renal tubular lesion and function loss in AAN through inhibition of renal activation of NLRP3 inflammasome. Our findings not only revealed a novel molecular mechanism responsible for the development and progression of AAN, but also pointed out potential therapeutics for AA-induced nephropathy by targeting renal NLRP3 inflammasome activation, which is of great significance to the prevention and treatment of AAN.

The dysfunction of NLRP3 inflammasome has been increasingly be reported to participate in the development and progression of a series of kidney diseases, including acute kidney injury (12, 29), chronic kidney disease (30), diabetic nephropathy (31, 32), as well as autoimmune kidney disease (33–35). In recent years, it has been intensively reported that NLRP3 inflammasome/Caspase-1/IL-1β signaling pathway was involved in renal tubular lesion during acute kidney injury (36–38). In this study, we found for the first time that NLRP3 inflammasome was aberrantly activated in AAN with elevated NLRP3 expression mainly localized in damaged renal tubules. Notably, NLRP3 overexpression was largely co-localized with injured renal tubules, indicating a possible causal link between elevated NLRP3 expression and aggregated tubular injury. However, it should be noted that our study focused just on NLRP3 inflammasome activation in renal tubules (tubular inflammation). Further investigations into tubulointerstitial inflammation (39, 40) will provide a more comprehensive view of renal inflammatory responses in AAN. Although we elucidated that activation of NLRP3 inflammasome was abnormally induced both *in vitro* and *in vivo* after AA exposure, it should be noted that the exact molecular mechanism for the initiation of NLRP3 inflammasome activation in the progression of AAN remains unclear. Moreover, it is generally recognized that NLRP3 inflammasome activation in sterile inflammatory diseases is initiated by recognition of endogenous or exogenous danger signals released from injured kidney tissue such as potassium efflux, extracellular ATP, reactive oxygen species, mitochondrial stress, and endoplasmic reticulum stress (15, 41, 42). Therefore, in-depth investigations are still needed to clarify the initial priming steps for NLRP3 inflammasome activation in AA-induced nephropathy.

In the current study, we also found that renal tubular injury in AAN was markedly alleviated in mice with NLRP3 or Caspase-1 deficiency and thus demonstrated that renal NLRP3 inflammasome activation probably participated in the onset and progression of AAN. Although the molecular mechanism underlying the pathogenesis of AAN has been intensively investigated in recent studies (43), the role of NLRP3 inflammasome has not been previously described in AA-induced nephropathy. Notably, we revealed for the first time that renal NLRP3 inflammasome activation probably contributed to the pathogenic mechanism of AAN, therefore identifying a novel correlation between NLRP3 inflammasome activation and the progression of AAN. Nevertheless, it should be pointed out that a more detailed characterization of essential components for NLRP3 inflammasome activation in diverse compartments within renal tissue such as epithelium, endothelium, and interstitium will further benefit the understanding of AA-induced nephropathy. Given that exposure of renal tubular epithelial cells to AA induced an obvious activation of NLRP3 inflammasome, NLRP3 inflammasome activation may also occurred in immunocytes such as infiltrated macrophages during the development and progression of AAN. Cell-specific knockdown of the NLRP3 inflammasome is required to further address the role of NLRP3 inflammasome activation in tubular epithelial cells in AA-induced nephropathy (44). Moreover, future studies should also explore the crosstalk between tubular epithelial cells and other cell types especially inflammatory cells to mediate renal tubular injury in AAN.

IL-22 has been intensively reported to play a critical role in various kidney diseases. It has been demonstrated that IL-22 specifically augmented tubular cell integrity and epithelial barrier function in chronic obstructive nephropathy (45). Furthermore, IL-22 ameliorated renal injury and accelerated tubular regeneration and recovery by targeting proximal tubule epithelium in acute kidney injury (18, 19). Although mounting evidence implicated the renoprotective potential of IL-22 in various kidney diseases, the role of IL-22 in AAN has never been described. In this study, we indicated that administration of IL-22 markedly attenuated renal tubular injury and function loss in AA-induced nephropathy. Given that previous studies indicated that IL-22 could promote proliferation, survival and repair of epithelial cells in diverse tissues and the IL-22 receptor was exclusively expressed by renal tubular epithelial cells in renal tissues, it is not surprising that IL-22 has a potent effect on renal protection and regeneration by targeting tubular epithelial cells. Notably, we found that IL-22 possessed potentials to ameliorate renal fibrosis in the short-term mouse model of AAN (25). However, it remains controversial whether IL-22 exerted pro-fibrotic or anti-fibrotic effects in renal diseases as well as other fibrotic disorders (20, 45–47). The mouse model of chronic AAN (40, 48) should be used to further validate the nephroprotective and anti-fibrotic effects of IL-22. Interestingly, IL-22 suppressed renal tubular injury in AAN through suppression of NLRP3 inflammasome activation, indicating that suppression of NLRP3 inflammasome-driven inflammatory response in AAN is critical to preventing tissue damage and preserving renal function. The blockade of persistent and excessive sterile inflammatory responses with specific antagonists of NLRP3 inflammasome could be a promising therapeutic strategy for the prevention or treatment of the adverse outcomes associated with kidney injury. However, it should be noted that the activation of NLRP3 inflammasome is a two-faced biological mechanism, ultimately leading to dangerous stimuli removal or irreversible tissue damage. Therefore, the appropriate regulation of the NLRP3 inflammasome is needed to apply to patients with renal inflammatory disorders. It has been reported that IL-22 could promote NLRC4 activity for sustainable production of IL-1Ra and thus restrained pathogenic NLRP3 inflammasome activity (24), which could possibly be the underlying mechanism for the suppression of NLRP3 inflammasome by IL-22. Particularly, to further verify IL-22 as a novel therapeutic agent against AAN by targeting NLRP3 inflammasome activation, more efforts should be devoted to exploring the possible crosstalk between the IL-22 pathway and the NLRP3 inflammasome signaling in order to decipher exactly how IL-22 regulates NLRP3 inflammasome activation.

In summary, the present study not only revealed the pathogenic role of NLRP3 inflammasome activation in AA-induced nephrotoxicity, but also highlighted the anti-inflammatory properties of IL-22 through downregulation of NLRP3 inflammasome activation in AAN. These findings strongly indicated that targeting NLRP3 inflammasome activation or blocking its downstream effectors may be a promising therapeutic strategy for AA-induced nephropathy.

## Data Availability Statement

All datasets generated for this study are included in the manuscript/supplementary files.

## Ethics Statement

All the *in vivo* experimental procedures were performed according to the standards approved by Animal Ethical Committee of School of Pharmacy at Fudan University.

## Author Contributions

SW, JF, and XM designed the research, performed the experiments, analyzed the data, and wrote the manuscript. JL, YL, XZ, WC, and YW performed the experiments and analyzed the data. GM contributed to data interpretation and intellectual property. DJ contributed to conception and design, interpretation of the data, and acquiring of the funding.

### Conflict of Interest

The authors declare that the research was conducted in the absence of any commercial or financial relationships that could be construed as a potential conflict of interest.
